# Three-Dimensional Bone Morphology for Identifying Risk Factors for Jones Fractures using Statistical Shape Modeling: A Retrospective Study

**DOI:** 10.21203/rs.3.rs-7555791/v1

**Published:** 2025-09-17

**Authors:** Takuma Miyamoto, Hiroaki Kurokawa, Tomohiro Matsui, Yuki Ueno, Norihiro Tsujimoto, Munehiro Ogawa, Akira Taniguchi, Amy L. Lenz, Yasuhito Tanaka

**Affiliations:** Nara Medical University; Nara Medical University; Takanohara Central Hospital; Nara Medical University; Nara Medical University; Nara Medical University; Nara Medical University; University of Utah; Nara Medical University

**Keywords:** computed tomography analysis, fifth metatarsal stress fracture, Jones fracture, statistical shape modeling, three-dimensional bone morphology

## Abstract

**Background:**

Jones fractures are fifth metatarsal stress fractures frequently seen in athletes and often lead to delayed union, non-union, and refracture. Identifying risk factors for Jones fractures is essential for prevention. While two-dimensional (2D) imaging has provided insights, it cannot adequately assess three-dimensional (3D) bone morphology. Statistical shape modeling (SSM) enables comprehensive 3D evaluation of anatomical variations, although its role in Jones fractures remains unclear. This study aimed to identify 3D morphological factors associated with Jones fractures using SSM and assess postoperative morphological changes.

**Methods:**

In this retrospective comparative study, we analyzed 20 patients with Jones fractures and 20 matched controls. All patients underwent headless compression screw fixation. Computed tomography was used to create 3D models of the fifth metatarsal. Segmentation and alignment were performed, followed by SSM with ShapeWorks. Principal component analysis (PCA) identified shape variations. Statistical comparisons were made between preoperative fracture cases and controls and between preoperative and postoperative cases.

**Results:**

SSM identified six significant PCA modes, accounting for 78.3% of shape variation. The second mode showed significant differences between fracture patients and controls (p = 1.32e-04), demonstrating greater adduction of the metatarsal base, reduced articular surface, proximally extended tuberosity, and straighter, thicker shaft in fracture cases. No significant postoperative morphological changes were observed.

**Conclusion:**

Distinct 3D morphological characteristics of the fifth metatarsal–including base adduction, proximal tuberosity extension, and shaft straightening–may increase susceptibility to Jones fractures. The absence of postoperative changes suggests that surgical fixation does not alter these features.

## Background

Metatarsal stress fractures are a common type of stress injuries affecting bones of the lower extremity [1]. Jones fracture, a common sports foot bone injury, can occur in people of all ages and with different physical activity levels [2]. This type of fracture has a high incidence of delayed union, non-union [3–5], and refracture [6]. Consequently, Jones fractures may require an extended recovery period, sometimes preventing affected patients from returning to sport [3],[7]. It is important to accurately identify and understand risk factors for Jones fracture to prevent their occurrence.

Many studies have investigated risk factors for Jones fractures and evaluated anatomic [8–12], biological [13], and biomechanical features [3]. Historically, evaluating anatomical features using two-dimensional (2D) radiographic views has contributed to the identification of Jones fracture pathophysiology [8, 9, 12]. However, these 2D approaches are limited in accurately visualizing their three-dimensional (3D) morphology. Furthermore, conventional radiographs have inherent errors due to variations in rotational positioning during image acquisition [14].

Recently, high-resolution volumetric imaging (i.e., computed tomography [CT] and magnetic resonance imaging) has become a common tool for diagnosis and evaluation. Although CT images can provide 3D information, most often in clinical practice, these images are evaluated using the same or similar 2D measurements used for plain X-rays and conventional radiographs [15]. Statistical shape modeling (SSM) provides a 3D evaluation of population-wise anatomical variation and group-wise shape differences in anatomies reconstructed from volumetric imaging data [2, 12]. SSM can provide more accurate and comprehensive morphological evaluations of musculoskeletal tissue [16],[17]. However, its role in the assessment of Jones fractures remains unclear.

Herein, we aimed to identify risk factors for 3D fifth metatarsal bone morphology by comparing patients with Jones fractures sustained during sports activities with asymptomatic controls using SSM. We also examined whether 3D bone morphology is affected by surgery by comparing the preoperative and postoperative 3D fifth metatarsal bone morphologies of Jones fractures using SSM. We hypothesized that there would be differences in the 3D fifth metatarsal bone morphologies present in Jones fractures and that surgery would not significantly affect bone morphology. These study findings will provide insight into developing approaches to improve the management and treatment of Jones fractures.

## Methods

### Study Design

**Figure 1:**
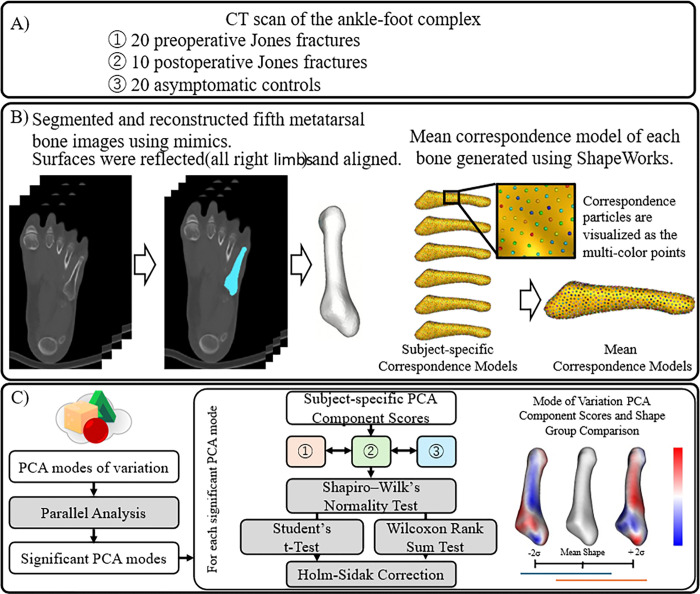
Computational study workflow. (a) Computed tomography scans of the study participants. (b) Three-dimensional reconstruction of the fifth metatarsal bone to develop statistical shape modeling and determine principal component analysis modes of variation. (c) Nonspurious principal component analysis modes were identified for each bone correspondence model using a parallel analysis followed by group comparisons of shape and principal component analysis component scores.

This retrospective comparative study was approved by the Institutional Review Board of our affiliated institutions. An opt-out statement regarding the application of medical data was published on our institute’s website. This study was performed in accordance with the principles of the World Medical Association’s Declaration of Helsinki. The study workflow is illustrated in Figure 1.

### Data Collection

This study included 27 patients who were diagnosed by radiography and underwent surgical treatment for Jones fractures sustained during sports activities at our institution between 2014 and 2024. Informed consent was obtained from all participants.

All patients underwent headless compression screw fixation. Exclusion criteria were prior surgeries on the same limb, the absence of preoperative CT data from the heel to the toe, and injuries resulting from non-sport-related activities. Ultimately, 20 patients were included in the analysis (mean age, 19.1 ± 2.54 years; mean height, 173 ± 5.15 cm; mean weight, 69.4 ± 15.0 kg; mean body mass index (BMI) of 23.0 ± 17.2 kg/m^2^). The study cohort comprised 19 male participants and one female participant. Regarding their sports histories, 14 participants had previously played soccer, two had played basketball, one had participated in rugby, one had participated in judo, one had participated in track and field, and one had played hockey. Postoperative CT scans were available for 10 of 20 patients. The 10 patients were male, were included in the postoperative group, and had a history of playing soccer. Furthermore, none of the patients had recurrent fractures, and all returned to their previous sport levels.

An asymptomatic control group comprising individuals who underwent CT scanning of the ankle-foot complex at our institution was also included. This group comprised 20 participants who matched the Jones fracture group in terms of age, height, weight, BMI, and sex. The control group had no history of ankle joint injury, deformity, or surgical trauma. The mean age of this group was 19.1 ± 2.54 years, with a mean height of 173 ± 5.15 cm, a mean weight of 69.4 ± 15.0 kg, and a mean BMI of 23.0 ± 17.2 kg/m^2^.

Herein, CT was performed using an OPTIMA 660 device (General Electric, Boston, MA, USA) with a 0.625-mm slice thickness and × 512/512 matrix resolution. For sample size calculations involving SSM, typical statistical methods of a priori sample size calculation were replaced by quantitative metrics of compactness, generalization, and specificity to assess the model outputs and optimization.

### Segmentation

For each participant, CT images were auto-segmented to create 3D models of the fifth metatarsal bone, and manual segmentation was performed to create a final model of the postoperative bone that completely excluded the effects of the screws (Mimics 26.0, Materialise, Leuven, Belgium). The generated bone surfaces were consistently meshed and smoothed using 3-matic 18.0 (Materialise). Preprocessing of the 3D bone reconstructions included mirroring the left foot to represent the right foot and aligning and centering using an iterative closest point algorithm [18].

### SSM

Single-domain SSM was performed for the 3D bone model, across all 40 participants, to generate statistical shape models using ShapeWorks 6.5.0 [19]. The methods used by ShapeWorks rely on particle-based shape models to place landmarks (i.e., correspondence particles) on the shapes using an optimization scheme [20]. The total particle count for the fifth metatarsal bone was 1,024. Corresponding particle locations were analyzed to define mean shapes and quantify bone shape differences across the population. The Procrustes algorithm was used to remove the scale from the shape model analysis [21]. Principal component analysis (PCA) was used to reduce the high-dimensional data (i.e., the location of all corresponding particles) to a small set of linearly uncorrelated components or modes of variation [19],[22, 23].

### Statistical Analysis

PCA modes containing significant variations were determined by parallel analysis [24]. Within significant PCA modes, PCA component scores were tested for normality using a Shapiro–Wilk test [25] and compared using the appropriate Student’s t-test (for normal) or Wilcoxon rank sum test (for non-normal) to compare PCA scores between patients with preoperative Jones fractures and control participants, as well as between preoperative and postoperative Jones fractures within that mode identified by parallel analysis. PCA component score tests across modes for a specific domain were corrected using a Holm–Sidak correction to reduce the probability of type 1 error [26, 27]. For all statistical measures, an alpha value of 0.05 was used (*p* < 0.05).

Statistical analyses were performed using MATLAB R2022a software. For each significant mode, the distance between the mean surface and ±2 standard deviation shapes was calculated and visualized using CloudCompare (v2.14. (www.cloudcompare.org).

## Results

Participants’ characteristics are summarized in Table 1.

**Figure 2:**
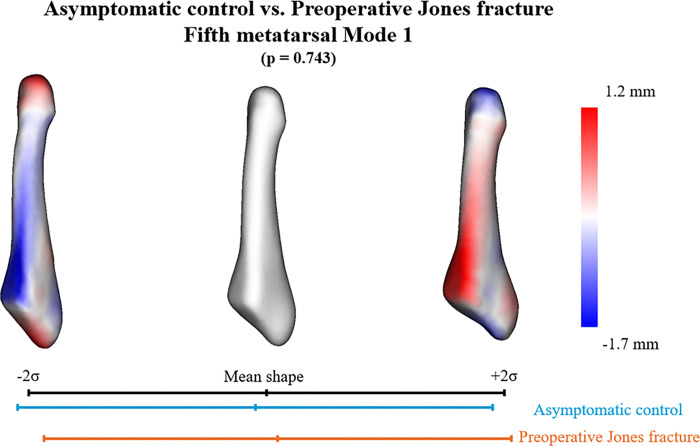
Principal component analysis (PCA) Mode 1. The quantitative visual description describes 32.5% of the explained variance and the PCA component score analysis. No significant difference was observed between asymptomatic controls and preoperative Jones fractures (*p* = 0.743).

**Figure 3:**
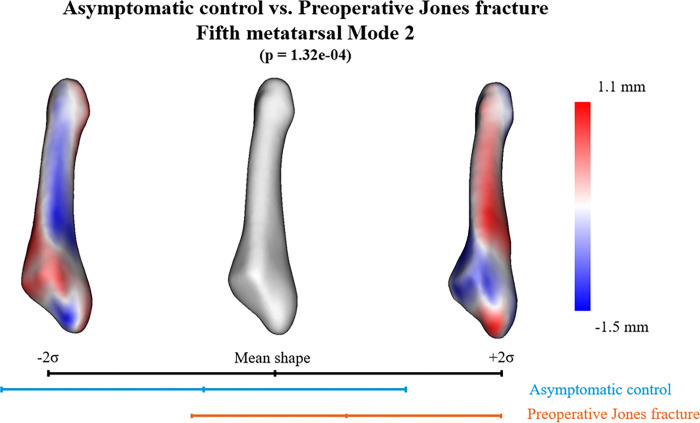
Principal component analysis (PCA) Mode 2. The quantitative visual description describes 22.0% of the explained variance and the PCA component score analysis. A significant difference was observed between asymptomatic controls and preoperative Jones fractures (*p* = 1.32e-4).

**Figure 4:**
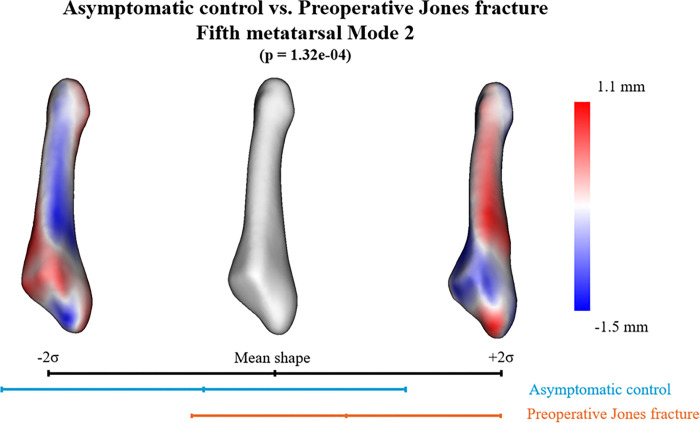
Principal component analysis (PCA) Mode 3. The quantitative visual description describes 8.1% of the explained variance and the PCA component score analysis. No significant difference was observed between asymptomatic controls and preoperative Jones fractures (*p* = 0.743).

In the SSM with asymptomatic controls and preoperative Jones fractures, six PCA modes were significant and described 78.3% of the overall shape variation. The six individual modes (1–6) contained 32.5%, 22.0%, 8.1%, 6.0%, 5.0%, and 4.6% significant variations, respectively (Table 2). Anatomical variations were observed across significant modes. However, when comparing the mean shape parameters of the asymptomatic controls and preoperative Jones fractures for these modes, the second mode of variation was the only mode with significant differences (*p* = 1.32e-04) in the PCA component scores (Table 2). The first mode of variation primarily reflected differences in the length and thickness of the fifth metatarsal bone (Fig. 2). The second mode of variation, the only significantly different mode, primarily indicated that the group with preoperative Jones fractures had a smaller articular surface and steeper inclination at the fifth metatarsal base than the control group. These morphological differences resulted in the addition of a base relative to the diaphysis. Furthermore, the fifth metatarsal tuberosity extended more proximally, and the bone shaft was thicker and straighter in the group with preoperative Jones fractures (Fig. 3). The third mode of variation primarily demonstrated that as the bone shaft became thicker, the base became smaller, and the inclination of the articular surface became more gradual (Fig. 4). The fourth mode of variation demonstrated that as the tuberosity increased proximally, the curvature of the bone shaft became less pronounced. The fifth mode of variation demonstrated that as the curvature of the bone shaft became more pronounced, it became narrower. Finally, the sixth mode of variation demonstrated that as the tuberosity increased proximally, the curvature of the bone shaft became less pronounced.

**Figure 5:**
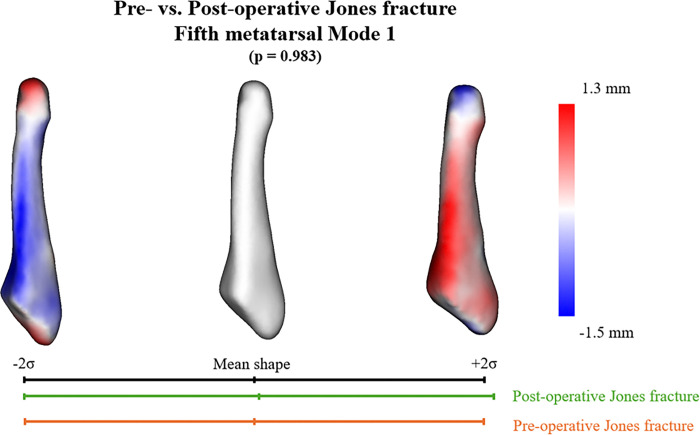
Principal component analysis (PCA) Mode 1. The quantitative visual description describes 34.3% of the explained variance and the PCA component score analysis. No significant difference was observed between preoperative and postoperative Jones fractures (*p* = 0.983).

Five PCA modes were significant in the SSM with preoperative and postoperative Jones fractures and described 70.6% of the overall shape variation (Table 2). No significant difference was observed when comparing the mean shape parameters of preoperative and postoperative Jones fractures in these modes. The first mode of variation primarily reflected the differences in the length and thickness of the fifth metatarsal (Fig. 5). In contrast, the second through fifth modes mainly represented differences in the curvature of the bone shaft and variations in the shape of the base, with minimal differences between the preoperative and postoperative conditions.

## Discussion

To the best of our knowledge, this study is the first to analyze differences in the 3D bone morphology of the fifth metatarsal between individuals with Jones fractures during sports activities and asymptomatic controls using SSM. Our findings indicate that individuals with Jones fractures exhibited greater adduction of the metatarsal base relative to the diaphysis than asymptomatic controls, resulting in a reduced articular surface and steeper inclination of the fifth metatarsal base. The fifth metatarsal tuberosity extended more proximally, and the bone shaft was thicker and straighter in individuals with Jones fractures than in those without. Furthermore, no significant differences were observed between the preoperative and postoperative fifth metatarsal morphologies. Previous investigations of the fifth metatarsal morphology in Jones fractures were based on 2D radiographic measurements [8, 9, 12], which are inherently affected by variations in foot positioning and rotational alignment at the time of imaging^[14]^. By leveraging 3D analysis, our study overcomes these limitations, providing a more precise characterization of bone morphology. These findings contribute to a deeper understanding of the pathogenesis of Jones fracture and may inform the development of preventive strategies.

We hypothesized that differences in the 3D fifth metatarsal bone morphology would be present in Jones fractures. Previous studies have reported that morphological characteristics of the fifth metatarsal may contribute to the risk of Jones fracture. Notably, an increased length of the fifth metatarsal has been identified as a significant risk factor for stress fractures of this bone [8],[9],[28]. Among these studies, the one by Fujitaka et al. [8] specifically reported that elongation is confined to the proximal portion of the fifth metatarsal. Karnovsky et al. [28] also reported that straight fifth metatarsals are associated with an increased risk of developing Jones fracture. The present study yielded similar findings, demonstrating that the fifth metatarsal tuberosity extended more proximally and the bone shaft was straighter. These findings supported the validity of our results and reinforced our hypotheses. Furthermore, studies investigating the relationship between metatarsal morphology and other anatomical characteristics have suggested that a reduced fourth-to-fifth intermetatarsal angle is associated with an increased risk of fifth metatarsal stress fractures [9, 28–30]. Although we did not investigate the relative relationships, we observed that Jones fractures exhibited greater adduction of the metatarsal base relative to the diaphysis, resulting in a reduction of the fourth-to-fifth intermetatarsal angle in 2D measurements. This observation suggests that the 3D evaluation of bone morphology provides more detailed insights than 2D measurements.

Our finding that Jones fractures exhibit greater adduction of the metatarsal base than that of the diaphysis is particularly intriguing. One limitation of this study is that the observed differences in 3D bone morphology between the Jones fracture and asymptomatic groups may represent a combination of causes and effects. This suggests that greater adduction of the metatarsal base relative to the diaphysis is a consequence rather than a cause of Jones fractures. If this was the case, the Jones fracture does not primarily result from dorsiflexion at the fracture site but rather from an adduction mechanism. Most previous studies have emphasized the dorsiflexion forces acting on the fifth metatarsal, likely because they rely on plantar pressure measurements [5, 15, 25]. However, combined finite element motion analysis has demonstrated that the fracture site experiences an adduction moment during sidestep cuts and a dorsiflexion moment during cross-step cuts [31]. Our findings highlight the significance of this adduction moment and underscore its potential role in the mechanics of Jones fracture. This insight may be critical in guiding future kinematic analyses.

Intramedullary screw fixation is the most commonly used surgical procedure for treating Jones fractures in athletes [11,16,21–23,29,30]. At our institution, Jones fractures are treated using headless compression screws. In the present study, all surgeries were performed using this technique; however, no significant differences in the 3D morphology of the fifth metatarsal were observed between the preoperative and postoperative Jones fractures. This finding is consistent with our hypothesis because this surgical technique generally does not involve fracture reduction. Additionally, because all postoperative patients in our study could return to sports without experiencing refracture, we could not assess the influence of these morphological findings on postoperative outcomes. However, the absence of morphological changes after surgery suggests that the postoperative bone morphology of the fifth metatarsal remains distinct from that of asymptomatic individuals. Further research is required to explore the clinical implications of these findings.

This study has several limitations. First, it is unclear whether the observed changes in bone morphology are a cause or consequence of a Jones fracture. Therefore, our findings reflect a combination of causative and resulting factors. Further research, including longitudinal studies, is required to clarify this relationship. Second, the number of postoperative cases was insufficient to assess the association between postoperative bone morphology and clinical outcomes. In Japan, weight-bearing radiographs are commonly used for the postoperative evaluation of Jones fractures owing to economic and ethical considerations, resulting in a limited number of cases requiring postoperative CT. In SSM analysis, imaging with thin-slice acquisition is crucial. However, obtaining such thin-slice images is challenging in most general hospitals, and collecting cases from multiple institutions difficult. Future studies with larger sample sizes are required to better understand the impact of postoperative morphological differences on clinical outcomes. Third, although all participants were highly active athletes, they competed in different sports. Therefore, we could not fully account for the potential variations in bone morphology related to sports-specific demands. A larger cohort study with extensive CT data is required to further investigate this factor.

## Conclusions

We analyzed differences in the 3D bone morphology of the fifth metatarsal between athletes with Jones fractures and asymptomatic controls using SSM. Our findings indicate that individuals with Jones fractures exhibit greater adduction of the metatarsal base relative to the diaphysis, a more proximally extended fifth metatarsal tuberosity, and a thicker and straighter bone shaft. Furthermore, no significant differences were observed between the preoperative and postoperative fifth metatarsal morphologies. Although these findings contribute to a better understanding of the pathophysiology of Jones fractures, further research is required. Nevertheless, these findings provide novel insights into the pathogenesis of Jones fractures and highlight the importance of 3D morphological assessment in understanding fracture risk and treatment outcomes.

## Figures and Tables

**Table 1.** Characteristics of participants

	Case (n)	Age (years)	Height (cm)	Weight (kg)	BMI (kg/m^2^)
Asymptomatic control	20	19.6 ± 2.69	171 ± 6.57	66.4 ± 9.48	22.8 ± 2.81
Jones fracture (preoperative)	20	19.1 ± 2.54	173 ± 5.15	69.4 ± 15.0	23.0 ± 17.2
Jones fracture (postoperative)	10	19.6 ± 2.93	174 ± 4.17	65.8 ± 6.63	21.6 ± 2.01
*p*-value (α = 0.05)		>0.05	>0.05	>0.05	>0.05

BMI: body mass index

**Table 2.** Parallel analysis results for each mode

		Mode 1	Mode 2	Mode 3	Mode 4	Mode 5	Mode 6
Control and preoperative	Eigenvalue	32.5%	22.0%	8.1%	6.0%	5.0%	4.6%
	*p*-value*	0.743	1.32e-04	0.743	0.135	0.424	0.424
Pre- and postoperative	Eigenvalue	34.3%	14.6%	8.8%	8.0%	5.0%	-
	*p*-value	0.983	0.983	0.865	0.983	0.983	-

* Denotes nonparametric data. The cell in green indicates a statistically significant value. For all measures, significance was set at *p* < 0.05. Eigenvalues taken from the compactness test reported as percentages of explained variance, along with normality test and principal component analysis component score results for each mode reporting significant differences between groups within a mode of variation

## Data Availability

The datasets used and/or analyzed during the current study are available from the corresponding author, Takuma Miyamoto, on request.

## Abbreviations

2D two: dimensional
3D three: dimensional
BMI: body mass index
CT: computed tomography
PCA: principal component analysis
SSM: statistical shape modeling

## References

[1] Sun J, Feng C, Liu Y, Shan M, Wang Z, Fu W, Niu W. Risk factors of metatarsal stress fracture associated with repetitive sports activities: a systematic review. Front Bioeng Biotechnol. 2024;12:1435807. 39175621 10.3389/fbioe.2024.1435807 PMC11338896

[2] Porter DA. Fifth metatarsal Jones fractures in the athlete. Foot Ankle Int. 2018;39:250–8. 29228800 10.1177/1071100717741856

[3] Kavanaugh JH, Brower TD, Mann RV. The Jones fracture revisited. J Bone Joint Surg Am. 1978;60:776–82. 701310

[4] Portland G, Kelikian A, Kodros S. Acute surgical management of Jones’ fractures. Foot Ankle Int. 2003;24:829–33. 14655886 10.1177/107110070302401104

[5] Zogby RG, Baker BE. A review of nonoperative treatment of Jones’ fracture. Am J Sports Med. 1987;15:304–7.3661809

[6] Wright RW, Fischer DA, Shively RA, Heidt RS Jr,, Nuber GW. Refracture of proximal fifth metatarsal (Jones) fracture after intramedullary screw fixation in athletes. Am J Sports Med. 2000;28:732–6. 11032233 10.1177/03635465000280051901

[7] Dameron TB Jr. Fractures and anatomical variations of the proximal portion of the fifth metatarsal. J Bone Joint Surg Am. 1975;57:788–92. 1099103

[8] Fujitaka K, Tanaka Y, Taniguchi A, Ogawa M, Isomoto S, Otuki S, Pathoanatomy of the Jones fracture in male university soccer players. Am J Sports Med. 2020;48:424–31. 31887064 10.1177/0363546519893365

[9] Lee KT, Kim KC, Park YU, Kim TW, Lee YK. Radiographic evaluation of foot structure following fifth metatarsal stress fracture. Foot Ankle Int. 2011;32:796–801. 22049866 10.3113/FAI.2011.0796

[10] Raikin SM, Slenker N, Ratigan B. The association of a varus hindfoot and fracture of the fifth metatarsal metaphyseal-diaphyseal junction: the Jones fracture. Am J Sports Med. 2008;36:1367–72. 18443278 10.1177/0363546508314401

[11] Williams DS 3rd, McClay IS, Hamill J. Arch structure and injury patterns in runners. Clin Biomech (Bristol). 2001;16:341–7. 11358622 10.1016/s0268-0033(01)00005-5

[12] Yoho RM, Carrington S, Dix B, Vardaxis V. The association of metatarsus adductus to the proximal fifth metatarsal Jones fracture. J Foot Ankle Surg. 2012;51:739–42. 22974812 10.1053/j.jfas.2012.08.008

[13] Smith JW, Arnoczky SP, Hersh A. The intraosseous blood supply of the fifth metatarsal: implications for proximal fracture healing. Foot Ankle. 1992;13:143–52. 1601342 10.1177/107110079201300306

[14] Krahenbuhl N, Lenz AL, Lisonbee R, Deforth M, Zwicky L, Hintermann B, Imaging of the subtalar joint: a novel approach to an old problem. J Orthop Res. 2019;37:921–6. 30638276 10.1002/jor.24220 PMC7311051

[15] Lenz AL, Lisonbee RJ. Biomechanical insights afforded by shape modeling in the foot and ankle. Foot Ankle Clin. 2023;28:63–76. 36822689 10.1016/j.fcl.2022.11.001 PMC10362888

[16] Bin Ghouth SG, Williams SA, Reid SL, Besier TF, Handsfield GG. A statistical shape model of soleus muscle morphology in spastic cerebral palsy. Sci Rep. 2022;12:7711. 35546597 10.1038/s41598-022-11611-z PMC9095689

[17] Pitocchi J, Plessers K, Wirix-Speetjens R, Debeer P, van Lenthe GH, Jonkers I, Automated muscle elongation measurement during reverse shoulder arthroplasty planning. J Shoulder Elbow Surg. 2021;30:561–71. 32707326 10.1016/j.jse.2020.07.007

[18] Schenker PS, BesI PJ, McKay ND. Method for registration of 3-D shapes. In: Sensor fusion IV: control paradigms and data structures. 1992. p. 586–606.

[19] Peterson AC, Lisonbee RJ, Krahenbuhl N, Saltzman CL, Barg A, Khan N, Multi-level multi-domain statistical shape model of the subtalar, talonavicular, and calcaneocuboid joints. Front Bioeng Biotechnol. 2022;10:1056536. 36545681 10.3389/fbioe.2022.1056536 PMC9760736

[20] Cates J, Elhabian S, Whitaker R. ShapeWorks. In: Statistical shape and deformation analysis. 2017. p. 257–98.

[21] Goodall C. Procrustes methods in the statistical analysis of shape. J R Stat Soc Ser B Methodol. 1991;53:285–321.

[22] Krahenbuhl N, Lenz AL, Lisonbee RJ, Peterson AC, Atkins PR, Hintermann B, Morphologic analysis of the subtalar joint using statistical shape modeling. J Orthop Res. 2020;38:2625–33. 32816337 10.1002/jor.24831 PMC8713294

[23] Lenz AL, Krahenbuhl N, Peterson AC, Lisonbee RJ, Hintermann B, Saltzman CL, Statistical shape modeling of the talocrural joint using a hybrid multi-articulation joint approach. Sci Rep. 2021;11:7314. 33795729 10.1038/s41598-021-86567-7 PMC8016855

[24] Rubén Daniel Ledesma PV-M. Determining the number of factors to retain in EFA. Practical Assessment, Research, and Evaluation 2007;12.

[25] Shapiro SS, Wilk MB. An analysis of variance test for normality (complete samples). Biometrika. 1965;52:591–611.

[26] Holm S. A simple sequentially rejective multiple test procedure. Scand J Stat. 1979;6:65–70.

[27] Šidák Z. Rectangular confidence regions for the means of multivariate normal distributions. J Am Stat Assoc. 1967;62:626–33.

[28] Karnovsky SC, Rosenbaum AJ, DeSandis B, Johnson C, Murphy CI, Warren RF, Radiographic analysis of national football league players’ fifth metatarsal morphology relationship to proximal fifth metatarsal fracture risk. Foot Ankle Int. 2019;40:318–22. 30403165 10.1177/1071100718809357

[29] Dixon S, Nunns M, House C, Rice H, Mostazir M, Stiles V, Prospective study of biomechanical risk factors for second and third metatarsal stress fractures in military recruits. J Sci Med Sport. 2019;22:135–9. 30057365 10.1016/j.jsams.2018.06.015

[30] O’Malley M, DeSandis B, Allen A, Levitsky M, O’Malley Q, Williams R. Operative treatment of fifth metatarsal Jones fractures (zones II and III) in the NBA. Foot Ankle Int. 2016;37:488–500. 26781131 10.1177/1071100715625290

[31] Miyazaki Y, Sugizaki R, Kawasaki M, Nakagawa T, Saho Y, Tateishi T. Fifth metatarsal strain distribution during cutting motions in soccer. Sports Biomech. 2023:1–17. 10.1080/14763141.2023.2241839 37533159

